# Data on rhizosphere pH, phosphorus uptake and wheat growth responses upon TiO_2_ nanoparticles application

**DOI:** 10.1016/j.dib.2018.02.002

**Published:** 2018-02-06

**Authors:** Rafia Rafique, Zahra Zahra, Nasar Virk, Muhammad Shahid, Eric Pinelli, Jean Kallerhoff, Tae Jung Park, Muhammad Arshad

**Affiliations:** a Institute of Environmental Sciences and Engineering, School of Civil and Environmental Engineering, National University of Sciences and Technology, Sector H-12, Islamabad 44000, Pakistan; b Atta-ur-Rehman School of Biological Sciences, National University of Sciences and Technology Islamabad, 44000, Pakistan; c Department of Environmental Science, COMSATS Institute of Information Technology, Vehari 61100, Pakistan; d EcoLab, Université de Toulouse, CNRS, l’Agrobiopôle, Toulouse 31326, France; e Department of Chemistry, Chung-Ang University, 84 Heukseok-ro, Dongjak-gu, Seoul 06974, Republic of Korea

**Keywords:** Rhizosphere pH, TiO_2_ NPs nanoparticles, Wheat, Phosphorus, Uptake

## Abstract

In this study, the data sets and analyses provided the information on the characterization of titanium dioxide nanoparticles (TiO_2_ NPs), and their impacts on rhizosphere pH, and soil-bound phosphorus (P) availability to plants together with relevant parameters. For this purpose, wheat (*Triticum aestivum* L.) was cultivated in the TiO_2_ NPs amended soil over a period of 60 days. After harvesting, the soil and plants were analyzed to examine the rhizosphere pH, P availability in rhizosphere soil, uptake in roots and shoots, biomass produced, chlorophyll content and translocation to different plant parts monitored by SEM and EDX techniques in response to different dosages of TiO_2_ NPs. The strong relationship can be found among TiO_2_ NPs application, P availability, and plant growth.

**Specifications Table**

**Table:** 

Subject area	*Environmental and agricultural applications*
More specific subject area	*Material synthesis, Effects of nanoparticles on soil-plant system,*
	*Nanobiotechnology*
Type of data	*Tables of TiO*_*2*_*effect on P concentration and plant biomass*
	*SEM and EDX images of TiO*_*2*_*NPs and their uptake by roots and leaves of plant*
	*Graphs of effect of TiO*_*2*_*NPs on rhizosphere soil, and P and chlorophyll content of leaves*
How data was acquired	*SEM, EDX, XRD, UV/Visible double beam spectrophotometer, chlorophyll meter (CCM 200-plus, Opti-Sciences, England, measurement area 0.7 cm*^*2*^*)*
Data format	*Raw, analyzed*
Experimental factors	*Wheat seeds were grown in sandy loam soil containing different concentrations of TiO*_*2*_*NPs. The experiments were held in a greenhouse for 60 days under ambient conditions.*
Experimental features	*Effects of soil application of TiO*_*2*_*NPs were measured on rhizosphere soil, roots, and shoots of plant*
Data source location	*Islamabad, Pakistan*
Data accessibility	*Data is available with this manuscript*

**Value of the data**

• The data provides information of TiO_2_ NPs effects on wheat over a period of 60 days for better understanding of their long-term impacts on plant growth.
• The data can help to understand the relationship between TiO_2_ NPs application and phytoavailability of P for farm and field level applications to ensure nutrient management.
• The data suggested the scientific community to extend the exposure time and comparison with other plant species instead of very short term bioassays.
• Future experiments can be compared with this data to predict the optimum concentrations of NPs for better plant development for different plant species.

## 1 Data

The datasets and analyses described the impacts of soil applied titanium dioxide nanoparticles (TiO_2_ NPs) on wheat (*Triticum aestivum* L.) plants. Corresponding figures, graphs, and images are provided with this article.

## 2 Experimental design, materials, and methods

### 2.1 Synthesis and characterization of TiO_2_ NPs

**Fig. 1:**
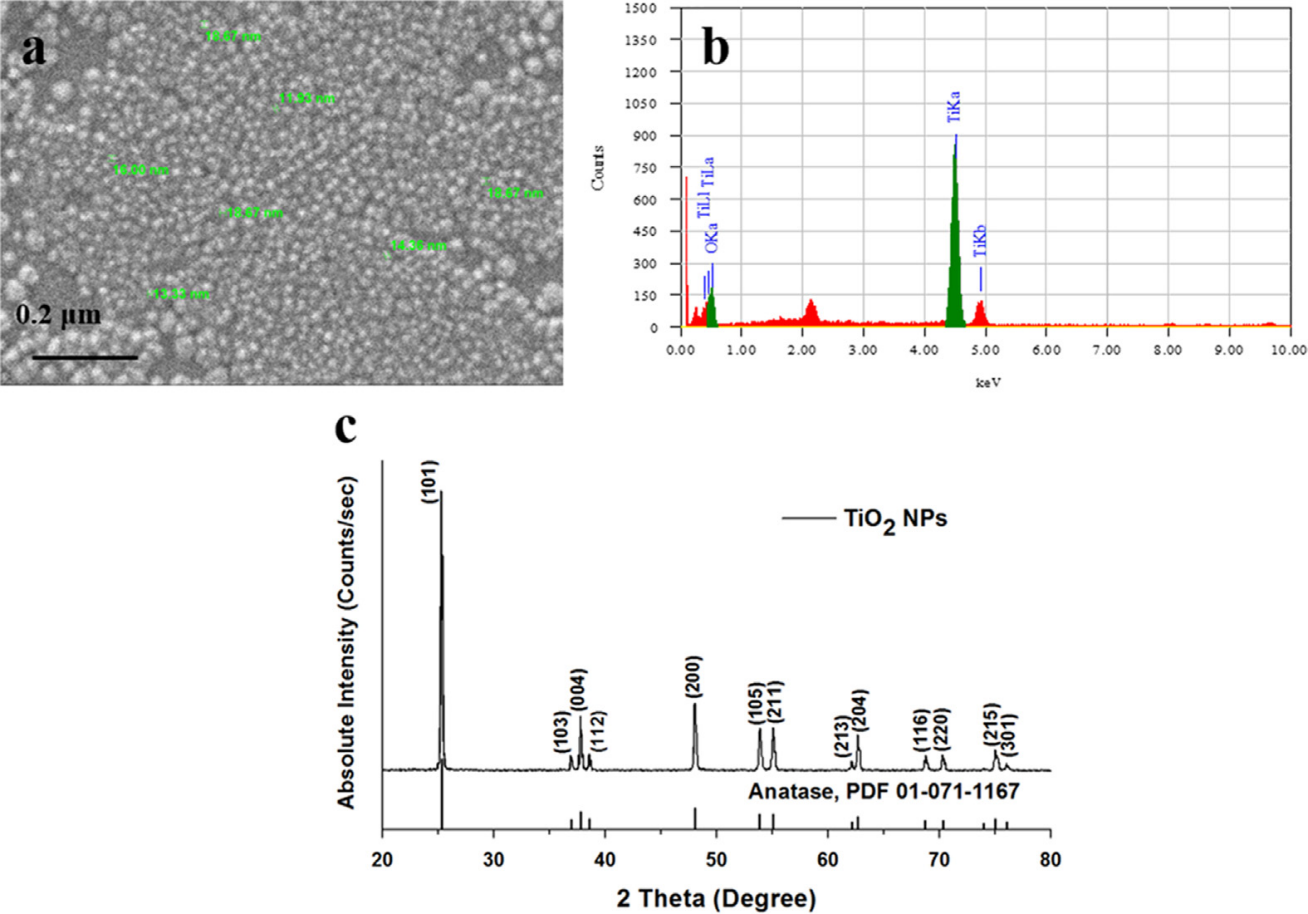
Characterization results of TiO_2_ NPs. (a) SEM image, (b) EDX and, (c) XRD spectrum of TiO_2_ NPs.

TiO_2_ general purpose reagent was obtained from Sigma Aldrich Inc. (purity > 99%, St. Louis, MO, USA) and further processed, and calcined at 500 °C to synthesize pure anatase crystal structure of TiO_2_ NPs as described in Zahra et al. [1]. Scanning electron microscope (SEM, Jeol, JSM 6490A, Tokyo, Japan), energy-dispersive X-ray spectroscopy (EDX, Jeol, JED 2300), and X-ray diffraction (XRD) analyses of as-prepared TiO_2_ NPs were performed as shown in Fig 1.

### 2.2 Soil application of TiO_2_ NPs

TiO_2_ NPs suspensions were prepared by mixing their various concentrations (0, 20, 40, 60, 80, and 100 mg L^−1^) in deionized water and sonicated for 30 min. Four replicates of each treatment level, and the control group (without TiO_2_ NPs) were maintained. Healthy seeds of wheat (Galaxy 2013) were obtained from the Ayub Agricultural Research Institute, Faisalabad, Pakistan. Three seeds were sown in each pot with as-prepared concentrations of TiO_2_ NPs. The experiments were conducted in a greenhouse for 60 days following randomized block design where the position of the pots was altered to avoid environmental bias effects.

### 2.3 Analysis of soil and plants

**Fig. 2:**
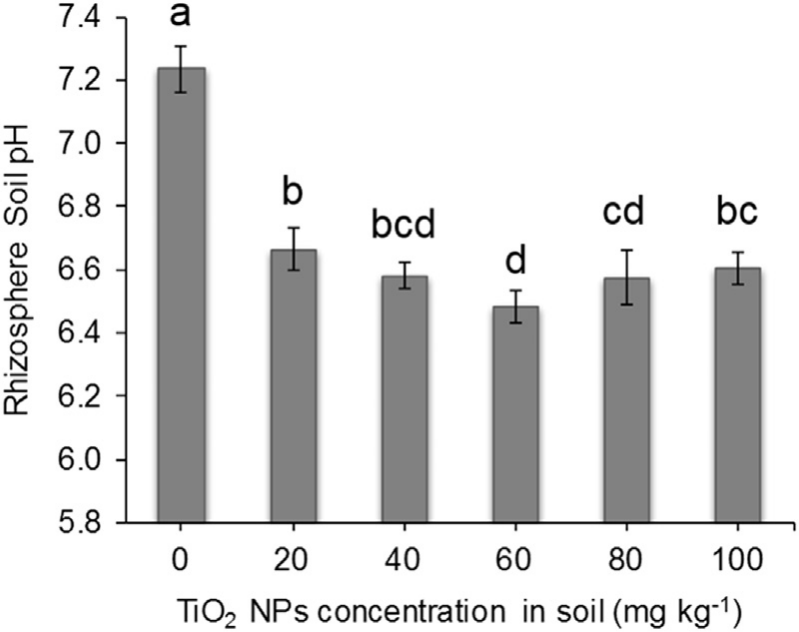
Effect of TiO_2_ NPs treatments on rhizosphere soil pH. Different alphabets correspond to statistically significant results at *p < 0.05*.

**FIG. 3:**
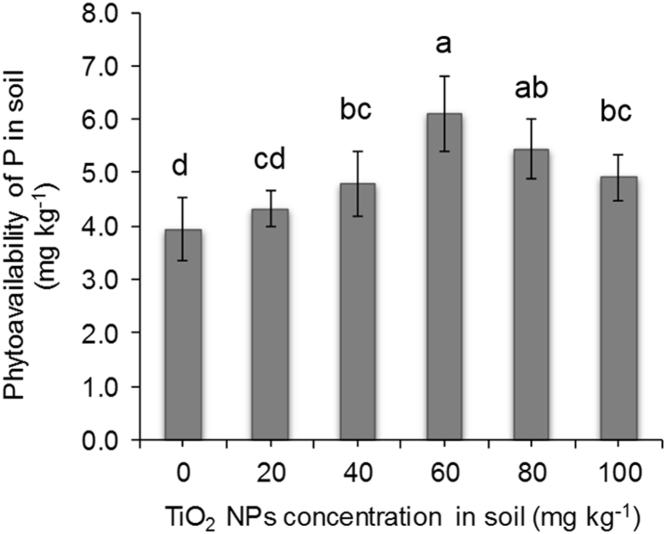
Effect of TiO_2_ NPs treatments on phytoavailability of P in rhizosphere soil. Different alphabets correspond to statistically significant results at *p < 0.05*.

After 60 days of TiO_2_ NPs exposure, the plants were uprooted and shaken carefully to remove soil at harvesting. The loosely bound soil adhered to the roots was collected with gentle washing in distilled water (100 mL) to investigate the rhizosphere pH (Fig. 2) and P (Fig. 3) using Olsen's method [2]. The roots and shoots were cut and dried in hot air oven for 48 h at 70 °C. After that, the dry biomass was recorded (Table 1) and stored for P analysis. For plant P content analysis, 100 mg of ground plant samples were added to acid mixture containing 5 mL of HNO_3_/HClO_4_ (2:1). This was digested on a hot plate followed by filtration through Whatman filter paper no. 42 to get clear aliquots for P content analysis (Table 2) using the vanado-molybdo-phosphoric acid colorimetric method [3].

**Table 1:** Effect of TiO_2_ NPs treatments on plant shoot and root dry biomass of wheat.

TiO_2_ NPs Concentration (mg kg^−1^)	Shoot dry biomass (mg)	Root dry biomass (mg)	Total dry biomass (mg)
0	0.73 ± 0.09a	1.11 ± 0.16a	1.37 ± 0.06a
20	0.89 ± 0.05b	1.47 ± 0.12b	1.70 ± 0.05b
40	0.73 ± 0.42b	1.30 ± 0.74b	1.71 ± 0.17b
60	0.63 ± 0.59b	1.19 ± 1.14b	1.81 ± 0.39b
80	0.95 ± 0.05b	1.65 ± 0.13b	1.84 ± 0.04b
100	0.91 ± 0.05b	1.52 ± 0.1b	1.71 ± 0.07b

The values are the means of four replicates ± Standard Deviation (SD). The means followed by similar letter (a) in the same column are not significantly different whereas (b) represents statistically significant difference at *p* < 0.05.

**Table 2:** Effect of TiO_2_ NPs treatments on plant shoot and root P concentration of wheat.

TiO_2_ NPs Concentration (mg kg^−1^)	Shoot P concentration (mg)	Root P concentration (mg)	Total P concentration (mg)
0	1.52 ± 0.17a	1.48 ± 0.13a	3.00 ± 0.03a
20	1.65 ± 0.08b	1.84 ± 0.12b	3.49 ± 0.14b
40	1.77 ± 0.06b	2.08 ± 0.16b	3.85 ± 0.22b
60	0.85 ± 0.08b	2.52 ± 0.19b	4.37 ± 0.47b
80	1.73 ± 0.07b	1.99 ± 0.18b	3.72 ± 0.18b
100	1.68 ± 0.06b	1.90 ± 0.16b	3.58 ± 0.15b

Data is the mean of four replicates ± Standard Deviation (SD). Means followed by different letters in the same column indicate significantly significant results at *p* < 0.05.

### 2.4 Estimation of leaf chlorophyll content

**Fig. 4:**
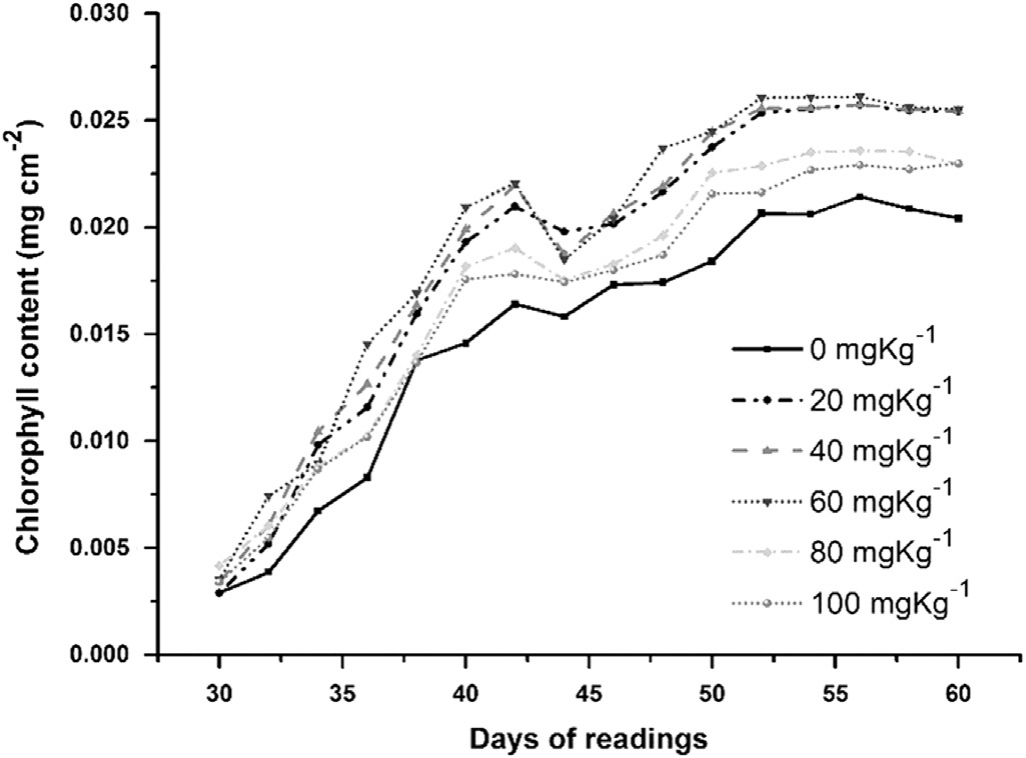
Effect of TiO_2_ NPs treatments on foliar chlorophyll content of wheat on daily basis.

A hand-held chlorophyll meter was used to measure the chlorophyll content index (CCI). The CCI readings were taken after the 30th day of NPs exposure for 16 alternate days until harvest. The everyday measurements are the mean of 32–48 readings for each treatment (Fig. 4). Following calibration Eq. (1) was used to process the raw data and convert the CCI index values to chlorophyll content expressed as m cm^−2^ [4].

(1) $$\text{y}\;\text{ = }\;\text{–}\;\text{2.20}{\text{e}}^{\text{-03 }}\;\text{+}\;\text{3.09}{\text{e}}^{\text{-03}}\;\text{x}\;\;\text{ – }\;\text{5.63}{\text{e}}^{\text{-05}}{\text{x}}^{\text{2}}$$

where y = Total chlorophyll content

x=Chlorophyllmetervalue

### 2.5 Microscopic analysis of plant

**Fig. 5:**
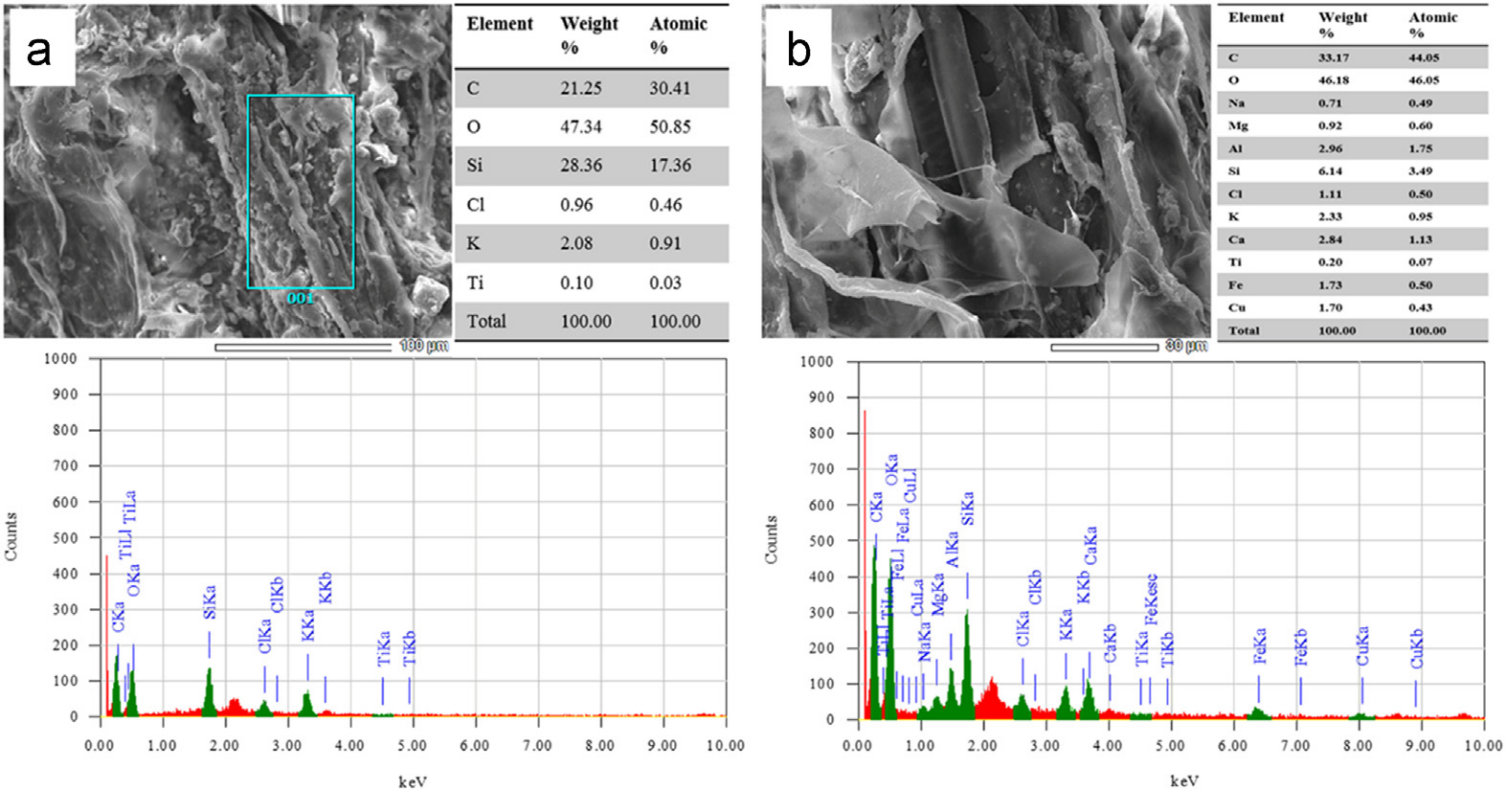
SEM and EDX analysis of wheat roots at (a) 0 mg kg^−1^, and (b) 60 mg kg^−1^. The EDX spectrum was measured at 20 keV.

**Fig. 6:**
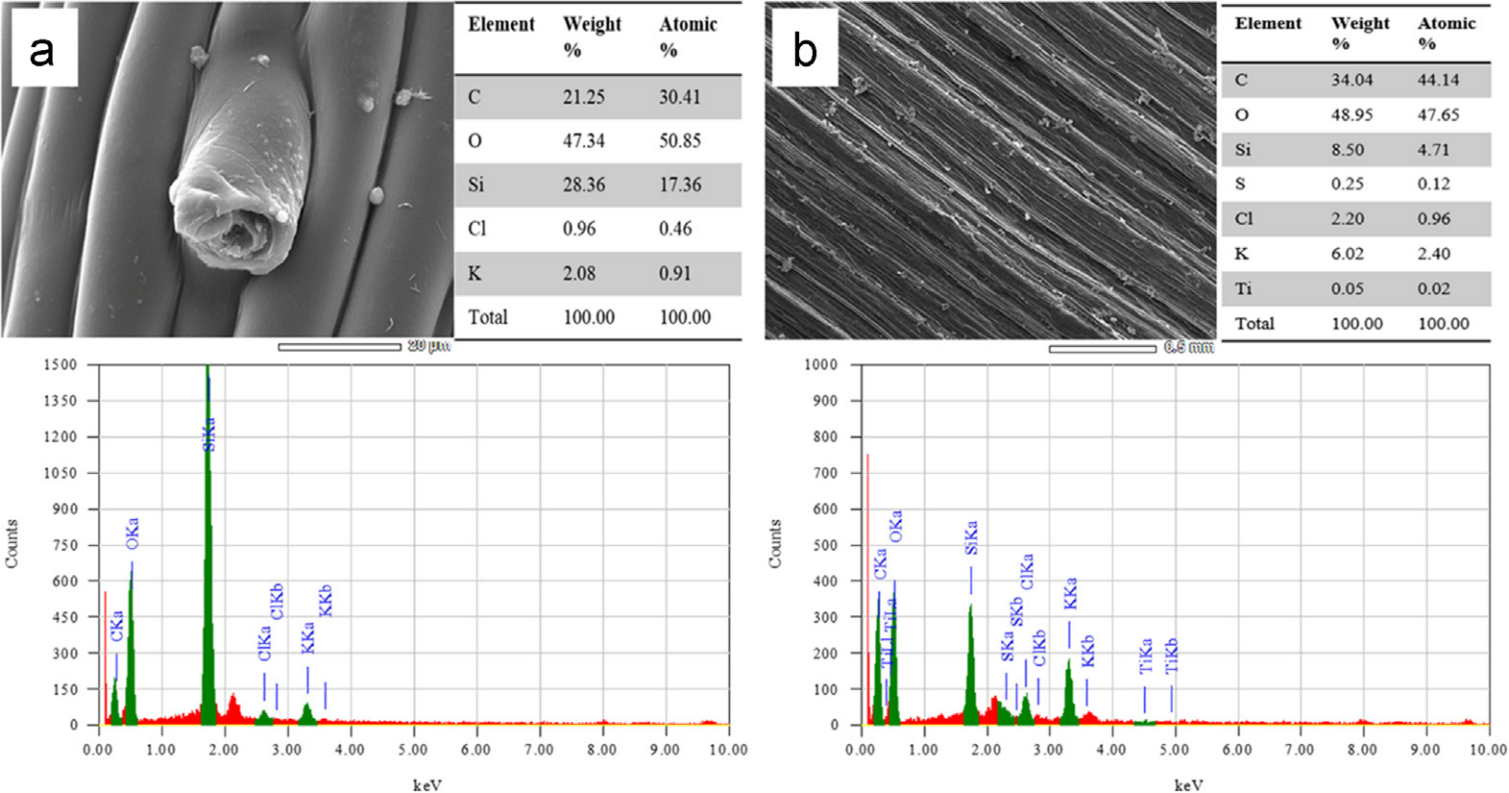
SEM and EDX analysis of wheat leaves at (a) 0 mg kg^−1^, and (b) 60 mg kg^−1^ of TiO_2_ NPs treatment. The EDX spectrum was measured at 20 keV.

To investigate the uptake of TiO_2_ NPs, plant samples were observed under SEM equipped with EDX to demonstrate the elemental composition of the control (0 mg kg^−1^ TiO_2_ NPs) and treated (60 mg kg^−1^ TiO_2_ NPs) samples of roots (Fig. 5), and shoots (Fig. 6).

### 2.6 Statistical analysis

The statistical significance analysis was done using Student's *T*-Test available in the Microsoft Excel analysis tool box. One-way ANOVA test was performed to identify statistically significant differences between the treatments. Statistix 8.1 was used to identify the least significant differences (LSD) at *p* < 0.05. All the data presented here supports the findings and discussion in Rafique et al. [5].
